# Evaluating Written Patient Information for Eczema in German: Comparing the Reliability of Two Instruments, DISCERN and EQIP

**DOI:** 10.1371/journal.pone.0139895

**Published:** 2015-10-06

**Authors:** Megan E. McCool, Josepha Wahl, Inga Schlecht, Christian Apfelbacher

**Affiliations:** Department of Medicine, Institute for Epidemiology and Preventive Medicine, University of Regensburg, Regensburg, Germany; University of Stellenbosch, SOUTH AFRICA

## Abstract

Patients actively seek information about how to cope with their health problems, but the quality of the information available varies. A number of instruments have been developed to assess the quality of patient information, primarily though in English. Little is known about the reliability of these instruments when applied to patient information in German. The objective of our study was to investigate and compare the reliability of two validated instruments, DISCERN and EQIP, in order to determine which of these instruments is better suited for a further study pertaining to the quality of information available to German patients with eczema. Two independent raters evaluated a random sample of 20 informational brochures in German. All the brochures addressed eczema as a disorder and/or therapy options and care. Intra-rater and inter-rater reliability were assessed by calculating intra-class correlation coefficients, agreement was tested with weighted kappas, and the correlation of the raters’ scores for each instrument was measured with Pearson’s correlation coefficient. DISCERN demonstrated substantial intra- and inter-rater reliability. It also showed slightly better agreement than EQIP. There was a strong correlation of the raters’ scores for both instruments. The findings of this study support the reliability of both DISCERN and EQIP. However, based on the results of the inter-rater reliability, agreement and correlation analyses, we consider DISCERN to be the more precise tool for our project on patient information concerning the treatment and care of eczema.

## Introduction

Eighty percent of patients actively seek information about how to cope with their health problems [1]. Having access to various information allows patients to take an active role in the medical decision making process [2]. While the majority of patients cite their doctor as the most important source of health information, 33% of patient use the Internet as a further source and 25% look for information in leaflets and books [1].

As patients’ access to information in health and medicine increases, the question of the quality of this information becomes ever more important [2]. Quality, in this case, refers to a number of domains including the accuracy of the information (is it evidence-based?), the presentation of information (is it clear and comprehensible for the patient?), and the recentness of the information (how up-to-date is this information?) [2,3]. Further considerations in terms of quality may encompass possible conflicts of interest (who is the publisher?), the qualification of the author (who wrote this?), and the expressed mention of other resources for patients (where else can patients turn to for information?). Ultimately, the leaflet, book or website should enable the patient to make a balanced, informed decision about the medical matter at hand.

To date, more than 500 instruments have been developed to evaluate the quality of patient information [3]. Some of these tools include the International Patient Decision Aids Standards (IPDAS) checklist [3], the JAMA benchmarks [4], Health on the Net (HON) code [5], DISCERN (no acronym) [6], and Ensuring Quality Information for Patients (EQIP) [7]. These tools can be applied to printed material or websites in order to assess the quality of the health information which is provided. As with any measurement tool, patient information assessment tools need to be valid (i.e. measure what they purport to measure) and reliable (i.e. measurements need to be free from measurement error as much as possible).

As part of a larger study, we were interested in examining the quality of written information available to patients in Germany who suffer from eczema (atopic eczema or atopic dermatitis). To this end, we took a closer look at two commonly used instruments: DISCERN and EQIP. Both instruments have been validated [6,7]. The aim in this study was to find the more reliable tool for assessing patient information on eczema in the German language. Reliability can be defined as the extent to which a scale or tool yields the same results on repeated use under the same conditions [8]. Important measures of reliability are known to be inter-rater correlation, intra-rater correlation and agreement [8]. Based on the reliability of these tools in German, we would then be able to choose which instrument would be preferred for a further study on the content and quality of the brochures themselves.

## Materials and Methods

### Description of instruments

DISCERN is a validated instrument designed to help both patients and health professionals evaluate the quality of written information on treatment choices [6]. Since its inception in 1999, it has been used in over 50 published studies related to patient information about cancer, chronic illnesses and disorders e.g. anorexia or ADHD [9–13]. The instrument was expanded in 2008 to better assess online resources [14]. DISCERN has primarily been used in English-speaking countries, although it has also been used in German, French, Dutch, Chinese and Iranian settings as well [9,11,15–17]. An authorized German translation of DISCERN was published by the German Agency for Quality in Medicine in 2000 [18]. DISCERN is available in German at www.discern.de.

EQIP, developed as an alternative to DISCERN in 2004, is another validated instrument which helps health professionals judge the quality of written information—both in print and online [7]. EQIP too has been used in studies which evaluate information on therapy options and/or chronic illnesses [19–21]. Due to its initial development in a pediatric setting, EQIP considers the informational needs which may be relevant for parents and caretakers of children [7], further underlining its appropriateness for our study of eczema care. Unlike DISCERN, EQIP evaluates the aspects of design and language as well. The instrument was expanded in 2008 in order to fulfill the British Medical Association patient information appraisal form [20,22]. EQIP is available in English at www.centralcancernetwork.org.nz/file/fileid/30657. The original instrument in English was translated into the German language by a native German speaker and back-translated by a native English speaker.

### Study design

The study took place in Regensburg, Germany and focused on informational brochures on eczema that were written in German. In March 2012 the study team contacted a total of 107 medical practices and 46 pharmacies in the city of Regensburg by mail (n = 153). The practices and pharmacies were asked to send in patient information brochures which are available to patients or customers suffering from eczema. Thirty-six brochures were obtained. Twenty randomly selected brochures were then evaluated by two independent raters—rater A (MMC) and rater B (JW)–in order to test the reliability of the instruments DISCERN and EQIP. After a thorough briefing on the two instruments, each rater evaluated the 20 brochures using both DISCERN and EQIP. Additionally, rater A (MMC) evaluated the brochures in a second round in order to test intra-rater reliability. All brochures were evaluated in a random order.

### Raters and brochures

The two independent raters had backgrounds in public health and medicine and were provided samples of the instruments (in German), the original publications for DISCERN and EQIP as well as the related handbook/ guideline for their usage. The raters then met to discuss any ambiguities in the implementation of the two instruments. Both raters evaluated the brochures independently of one another and were not allowed to discuss or view each other’s scores until the completion of rater A’s second evaluation. Both raters were German-speaking.

The informational brochures were written in German and ranged in length from 4 to 48 pages. Fifteen publications described the illness (eczema) as well as one or more therapy options / medications. Four publications focused solely on a particular medication or therapy option, while one publication addressed the illness (eczema) among children. Of the 20 publications evaluated, 14 targeted audiences of eczema patients in general. Five publications were specifically designed for children and their caretakers. They provided drawings, tips for care and activities for children who suffer from eczema. One brochure addressed both audiences separately: adults with eczema and children with eczema. In terms of publishing and authorship, 18 out of 20 were published by pharmaceutical companies. Only two were published by doctors or organizations without any visible pharmaceutical association. A list of the 20 brochures can be found in the supporting information (S1 File).

### Design of instruments

The DISCERN instrument, consisting of 16 items, can be divided into three sections: reliability of the information (8 items), quality of information on treatment choices (7 items), and an overall, final grade (1 item). This instrument entails questions to evaluate the aim of the brochure, the relevance, accuracy and timeliness of the data, therapy options and their effect on quality of life, as well as the advantages, disadvantages and side effects of therapies. For scoring, DISCERN uses a 5-point Likert scale, ranging from no/does not fulfill criterion (1 point) to yes/fulfills criterion (5 points). No half scores are allowed. The final question (#16) is an overall score of the publication/brochure; here, only 1, 3 or 5 can be selected. The scores are added together at the end of the evaluation; the maximum number of points possible is 80.

The EQIP instrument is composed of 20 items evaluating the following aspects: aim of the brochure, the accuracy and timeliness of the data, therapy options and their effect on quality of life, as well as the advantages, disadvantages and side effects of therapies. Furthermore, seven items of the EQIP instrument (35%) evaluate the language (word length, jargon, etc.) and presentation / design of the brochure—aspects which are not addressed in DISCERN. For scoring, EQIP uses a 4-level scoring system: yes/fulfills criterion (1 point), partly/somewhat fulfills criterion (0.5 point), no/does not fulfill criterion (0 points), and not applicable. The scores are added together at the conclusion of the evaluation; the maximum number of points possible is 20.

Table 1 illustrates similarities and differences of the two instruments.

**Table 1:** Comparison of DISCERN and EQIP.

Aspects of Instrument	DISCERN	EQIP
Purpose	To enable patients and information providers to judge the quality of written information about treatment choices	To enable patient information managers and health care professionals to measure the presentation quality of all types of written health care information
Number of questions	16	20
Content (Number of questions)		
Aim of information	(2)	(1)
Accuracy	(1)	-
Timeliness / date of publication	(1)	(1)
Evidence	(1)	-
Relevance to patients	(1)	(1)
Therapy options	(2)	(2)
Side effects / risks	(1)	(1)
Advantages / disadvantages	(2)	(2)
Change in lifestyle (quality)	(1)	(1)
Layout	-	(3)
Language	-	(3)
Author, Publisher	-	(1)
Further resources	(1)	(2)
Other	Shared decision making (1) Non treatment (1), Overall score (1)	Integration of patients (1) Clarity of graphics (1)
Scoring system	1 = does not fulfill/5 = fulfills (1 to 5 points)	yes/somewhat/no/NA (1, 0.5, 0 points, NA)
Maximum sum	80	20
Validated version in English	Yes (1999)[6]	Yes (2004)[7]
Validated version in German	Yes (2000)[18]	No
Audience / users	Health professionals and patients	Health professionals
Scope of application	Print and online	Print and online

### Data analysis

Data analysis was performed using SPSS. The purpose of the study was to measure the reliability and the agreement of the two instruments when applied to informational brochures on eczema. Reliability was assessed using the intra-class correlation coefficient (ICC) which is a commonly-used statistical measure of reliability [23]. In addition to evaluating reliability, the agreement between the two raters was also measured using Cohen’s Kappa—a statistic which has already been used in validation studies for both DISCERN and EQIP [6,7,24,25]. Finally, the linear correlation of the raters’ scores for each instrument was investigated using Pearson’s correlation [26].

The raters entered the score for each item– 16 items for DISCERN and 20 items for EQIP—into an Excel spreadsheet. Each rater had his/her own Excel table. Each brochure had two sums: one for the DISCERN instrument on a scale of maximum 80 points and one for the EQIP instrument on a scale of maximum 20 points. These will be referred to as the “raw scores.” However since the denominator of the raw scores varied, the raw scores were transformed into percentages as well. These will be referred to as the “percentage scores.” The two Excel tables were imported into SPSS 19.0, the software used for the calculation of the following statistics.

First, intra- and inter-rater reliability were assessed. The intra-rater reliability was based on the results from rater A’s first and second evaluation of the brochures. The ICCs were computed using the raw scores from the first and second evaluation; a two-way mixed model with absolute agreement was applied. For the inter-rater reliability, the first evaluation from rater A and the first and only evaluation from rater B were compared. The ICCs were computed using the raw scores from each rater’s evaluation; a two-way mixed model with absolute agreement was applied here as well. The ICCs were interpreted using the following categories for reliability: 0.0 to 0.10 virtually none, 0.11 to 0.40 slight, 0.41 to 0.60 fair, 0.61 to 0.80 moderate, and >0.81 substantial [27].

Then, Cohen’s Kappa was used for testing the agreement between rater A and B for each item of the two instruments. For instance, for Item 1 on the DISCERN instrument, how well did rater A and rater B agree with each other when evaluating the 20 brochures? The scores for each item were tallied. A weighted kappa (quadratic) was calculated for each individual item (16 items for DISCERN, 20 items for EQIP). With these individual kappas, an average kappa for each instrument could be calculated. The kappa coefficients for agreement were interpreted using the following categories according to Fleiss: 0.0 to 0.40 poor, 0.41 to 0.75 fair to good, and >0.76 excellent [28].

For the correlational analysis, the strength of the correlation between the raters’ answers was evaluated. The percentage scores from rater A and the percentage scores from rater B were correlated for each instrument. For rater A, the average of the first and second evaluation was used. Bootstrapping was used to estimate the 95% confidence intervals. The correlation coefficient was interpreted using the following categories according to Cohen: 0.0 no relationship, 0.10 to 0.29 weak relationship, 0.30 to 0.49 moderate relationship, and >0.5 strong relationship [29].

## Results

### Intra-class correlation coefficients

Table 2 shows the findings from the assessment of intra- and inter-rater reliability. The intra-rater reliability for both instruments was ‘substantial’ with ICCs >81%. The inter-rater reliability however yielded more varied results. The reliability of DISCERN with 81.4% would be classified as ‘substantial’ whereas that of EQIP, 72.1%, was only ‘moderate’. Furthermore the 95% confidence interval for EQIP’s inter-rater reliability spanned over 50 percentage points.

**Table 2:** Intra- and inter-rater reliability.

Instrument	Intra-rater Reliability	Inter-rater Reliability
	% ICC (95% CI)	% ICC (95% CI)
DISCERN	89.4 (67.7–96.1)	81.4 (50.3–92.9)
EQIP	91.5 (79.9–96.6)	72.1 (33.5–88.8)

### Kappa coefficients

Table 3 shows results from the analysis of agreement. The average kappa for DISCERN and for EQIP revealed ‘fair to good’ agreement, whereby the kappa for DISCERN was notably higher than that of EQIP. Once again the 95% confidence intervals for EQIP were broader than those for DISCERN.

**Table 3:** Kappa values for each item for DISCERN and EQIP.

DISCERN		EQIP	
Item	k	Item	k
1 Aims clearly described	0.8936	1 Description of medical topic	0.3704
2 Aims achieved	0.6017	2 Use of medical jargon	0
3 Relevance	0.7236	3 Length of sentences	0.4758
4 Sources of information	0.5455	4 Addresses reader	0.1600
5 Date of publication	0.4167	5 Tone / style	-0.2121
6 Balance / bias	0.5022	6 Layout / design	0.4500
7 Support / other resources	0.9030	7 Illustrations / diagrams	0.2889
8 Grey areas of treatment	0.5282	8 Order of information	-0.1250
9 Description of treatment	0.4507	9 Space for notes	0.9231
10 Benefits of treatment	0.3429	10 Contact information	0.1667
11 Risks of treatment	0.7783	11 Date of publication	0.8864
12 Results of non-treatment	0	12 Author / producer	0.7305
13 Quality of life	0.1362	13 Inclusion of patients in creation of brochure	0.6429
14 Alternatives described	0.7596	14 Use of generic names	0.6857
15 Support for shared decision making	0.6712	15 Quality of life	0.5238
16 Overall score	0.6429	16 Support / other resources	0.8644
		17 Purpose described	-0.0417
		18 Benefits of treatment	0.7273
		19 Risks and side effects	0.7727
		20 Alternatives described	0.8000
Average k	0.5931	Average k	0.4589
(95% CI)	(0.477–0.709)	(95% CI)	(0.280–0.637)

### Correlation analysis

Pearson’s correlation revealed that the strength of the relationship between the raters’ scores for each instrument was strong. The raters’ scores for DISCERN (r = 0.882, P < 0.001, 95% CI 0.753–0.964) correlated slightly stronger than the scores for EQIP (r = 0.802, P < 0.001, 95% CI 0.571–0.939).

## Discussion

In evaluating information in German for patients with eczema, both DISCERN and EQIP demonstrated good intra-rater and inter-rater reliability. Furthermore, both instruments illustrated ‘fair to good’ agreement based on the kappa results. These results are also consistent with previous work. In 2009 Bachelor and Ohya used DISCERN to assess brochures and websites about eczema and asthma [25]. Among experts, the average kappa value for DISCERN was 0.53 [25]. In our study the average kappa for DISCERN was 0.59. In 2004 Moult et al. created and validated EQIP using information available in children’s hospitals [7]. Expert ratings were in ‘fair to good’ agreement with an average kappa of 0.55 [7]. In our study, EQIP yielded ‘fair to good’ agreement among raters with an average kappa of 0.46.

In our study, there were four items in EQIP which yielded a negative kappa or a kappa of 0. These were items pertaining to the use of medical jargon (#2), tone/style (#5), order of the information (#8), and if the purpose is described (#17). This indicates that the agreement between the raters is no better than would be expected by chance [30]. The lack of agreement on item 2 may be due to the fact that one rater had a medical background and the other a public health background, implying that “medical jargon/language” might be interpreted differently by these two raters. Items 5 and 8 resulted in low kappas in other validation studies of EQIP [20]. The lack of agreement for these items and possibly for item 17 as well may be attributed to the subjectivity of the questions. Item 12 in DISCERN yielded a kappa of 0 as well; this question addressed whether or not the brochure described repercussions of not treating the illness. None of the brochures stated specifically what would happen if the eczema were not treated, resulting in a 0 for all answers on item 12 of DISCERN.

Overall, the results for DISCERN and EQIP showed only minor differences. The ICC for inter-rater reliability, the kappa values and the strength of correlation were all superior for DISCERN. Yet, the 95% confidence intervals for all coefficients for the two instruments were overlapping. This is likely due to the small sample size (n = 20) and to the variation in scores. The narrow confidence intervals for the coefficients of inter-rater reliability, agreement and correlation indicate that DISCERN seems to yield more consistent measurements, making it a more precise tool than EQIP.

However, two shortcomings of both instruments were identified. Neither instrument explicitly addresses the readability of the brochures. In the development of DISCERN, the panel focused specifically on the content of consumer health information, rather than on presentation, style or readability [6]. EQIP has only one item (#3) which measures the length of the words in the information material, but it does not comprehensively assess readability either [7]. Thus, other tools which specifically address readability, such as the Flesch Reading Ease score, should be used alongside these two instruments [31]. The Flesch Reading Ease can be applied to the German language as well. Furthermore, both instruments require a degree of subjectivity [20,32]. While the manuals, publications and websites for these two instruments provide further information about their respective use, the subjectivity of each user cannot be completely ruled out.

Two shortcomings of the study should be mentioned. First, while the expanded EQIP version was created to incorporate online information, this 36-item version would have been suitable for our written information as well. The expanded version may have given different results than the original 20-item version. Secondly, we acknowledge that this study focused specifically on printed material—brochures or leaflets—and did not include online resources i.e. websites. Certainly, an exploration of websites on eczema therapy options would have yielded broader and possibly different results.

Finally, while over 500 tools are available to assess patient information, we selected two. Other tools, such as the IDPAS checklist [3], Steckelberg’s criteria [2] (in German), and Check-In [33] (in German) were among those also considered. However, the decision to use DISCERN and EQIP was based on pretests and the need for tools which provided a score and could therefore be compared.

This was the first time that EQIP was used in a German language setting. The study showed a strong correlation of the German version of EQIP with DISCERN which suggests that the German EQIP is a valid instrument. However, validation of EQIP in German was not the prime aim of this study. Further work would be needed to corroborate the validity of the German EQIP version.

## Conclusion

The findings of this study support the reliability of both DISCERN and EQIP. However, based on the results of inter-rater reliability, agreement and correlation, DISCERN was found to be more precise compared to EQIP. We therefore consider DISCERN to be the preferred tool for our project on patient information on the treatment and care of eczema.

## Supporting Information

S1 File List of Brochures (n = 20). List of the 20 brochures which were evaluated.
(DOCX)

S2 File Data Set DISCERN. Rater A and Rater B’s scores for the 20 brochures scores in raw numbers using the instrument DISCERN.
(SAV)

S3 File Data Set EQIP. Rater A and Rater B’s scores for the 20 brochures scores in raw numbers using the instrument EQIP.
(SAV)
