# Supplementary material for: Evaluating Written Patient Information for Eczema in German: Comparing the Reliability of Two Instruments, DISCERN and EQIP
Source: PLoS One. 2015 Oct 6;10(10):e0139895. doi: 10.1371/journal.pone.0139895 (PMC4595422; doi:10.1371/journal.pone.0139895)
Supplement: S1 File — List of the 20 brochures which were evaluated. (DOCX) [file pone.0139895.s001.docx]

**Supplementary File 1**

**List of Brochures (n=20)**

| **Nr.** | **Title** | **Title in English** | **Nr. of Pages** | **Target Audience** | **Author / Publisher** |
| --- | --- | --- | --- | --- | --- |
| 1 | Das geht mir unter die Haut | That really gets under my skin | 48 | Children and caretakers | Karen Crowe / Astellas |
| 5 | Lipi und Kara | Lipi and Kara | 19 | Children and caretakers | NA / La Roche-Possay |
| 8 | Atopisches Ekzem | Atopic eczema | 34 | Eczema patients in general | Prof. Dr. Beate Tebbe / Intendis Dermatologie GmbH |
| 9 | Neurodermitis | Neurodermatitis | 15 | Eczema patients in general | Prof. Dr. med. D. Abeck / Spirig Pharma |
| 24 | Atopische Dermatitis | Atopic dermatitis | 15 | Eczema patients in general | NA / Novartis |
| 26 | Echt cool, Mama - es juckt nicht mehr | Cool, Mom - It doesn’t itch anymore | 6 | Children and caretakers | Thomas Gilb / Allergika |
| 30 | Ausdruck über Neurodermitis | Print out on Neurodermatitis | 4 | Eczema patients in general | Prof. Dr. med. Eberhard Paul / NA |
| 38 | Neurodermitis | Neurodermatitis | 19 | Eczema patients in general | NA / Dermapharm |
| 39 | Gewusst wie - richtiges Cremen bei ND | Know-how – The right creme for ND | 4 | Eczema patients in general | NA / Infectopharma |
| 58 | Nimm mich mit & kratz mich | Take me with you and scratch me | 4 | Children and caretakers | NA / Avene Eau Thermale |
| 66 | Neurodermitis bei Kindern | Neurodermatitis in children | 4 | Children and caretakers | Dr. Markus Ball / Hans Karrer GmbH |
| 67 | Neurodermitis, Kortisonfrei behandeln | Neurodermatitis, treatment without cortison | 7 | Eczema patients in general | NA / Novartis |
| 68 | Neurodermitis Ratgeber | Neurodermatitis advice | 11 | Eczema patients in general | NA / Essex Pharma (Lisino) |
| 91 | STOP - Nicht kratzen! | STOP – Don’t scratch! | 4 | Eczema patients in general | NA / Stiefel Laboratorium GmbH |
| 94 | Haut Ruhe | Skin set at ease | 4 | Eczema patients in general | NA / EUBOS |
| 107 | Neurodermitis? Kontaktekzeme? Behandeln ohne Cortison | Neurodermatitis? Contact Eczema? Treatment without cortison | 4 | Eczema patients in general | NA / Chefaro |
| 119 | Trockene, raue Haut - Ratgeber | Dry, Raw Skin - Guidebook | 6 | Eczema patients in general | NA / Hermal Pharma |
| 130 | Trockene und zu ND neigende Haut | Dry and eczema-prone skin | 28 | Children and Adults with Eczema | Dr. Hubert Reich / Dr. August Wolff Arzneimittel |
| 157 | Ekzeme und Neurodermitis? | Eczema and Neurodermatitis? | 16 | Eczema patients in general | NA / Spitzner Arzneimittel |
| 158 | Neues und Bewährtes bei Neurodermitis | New and Proven [Techniques] with Neurodermatitis | 17 | Eczema patients in general | Dr. Ingolf Dürr / Deutsche Krankenhausgesellschaft |
